# Strong patterns of intraspecific variation and local adaptation in Great Basin plants revealed through a review of 75 years of experiments

**DOI:** 10.1002/ece3.5200

**Published:** 2019-04-26

**Authors:** Owen W. Baughman, Alison C. Agneray, Matthew L. Forister, Francis F. Kilkenny, Erin K. Espeland, Rob Fiegener, Matthew E. Horning, Richard C. Johnson, Thomas N. Kaye, Jeff Ott, John Bradley St. Clair, Elizabeth A. Leger

**Affiliations:** ^1^ Department of Natural Resources and Environmental Science University of Nevada Reno Nevada; ^2^ Department of Biology University of Nevada Reno Nevada; ^3^ Rocky Mountain Research Station USDA Forest Service Boise Idaho; ^4^ Pest Management Research Unit USDA‐Agricultural Research Service Northern Plains Agricultural Laboratory Sidney Montana; ^5^ Institute for Applied Ecology Corvallis Oregon; ^6^ Deschutes National Forest USDA Forest Service Pacific Northwest Region Bend Oregon; ^7^ Washington State University Pullman Washington; ^8^ Pacific Northwest Research Station USDA Forest Service Corvallis Oregon; ^9^ Present address: The Nature Conservancy Burns Oregon

**Keywords:** common garden, intraspecific variation, local adaptation, meta‐analysis, natural selection, phenotypic traits, reciprocal transplant, restoration

## Abstract

Variation in natural selection across heterogeneous landscapes often produces (a) among‐population differences in phenotypic traits, (b) trait‐by‐environment associations, and (c) higher fitness of local populations. Using a broad literature review of common garden studies published between 1941 and 2017, we documented the commonness of these three signatures in plants native to North America's Great Basin, an area of extensive restoration and revegetation efforts, and asked which traits and environmental variables were involved. We also asked, independent of geographic distance, whether populations from more similar environments had more similar traits. From 327 experiments testing 121 taxa in 170 studies, we found 95.1% of 305 experiments reported among‐population differences, and 81.4% of 161 experiments reported trait‐by‐environment associations. Locals showed greater survival in 67% of 24 reciprocal experiments that reported survival, and higher fitness in 90% of 10 reciprocal experiments that reported reproductive output. A meta‐analysis on a subset of studies found that variation in eight commonly measured traits was associated with mean annual precipitation and mean annual temperature at the source location, with notably strong relationships for flowering phenology, leaf size, and survival, among others. Although the Great Basin is sometimes perceived as a region of homogeneous ecosystems, our results demonstrate widespread habitat‐related population differentiation and local adaptation. Locally sourced plants likely harbor adaptations at rates and magnitudes that are immediately relevant to restoration success, and our results suggest that certain key traits and environmental variables should be prioritized in future assessments of plants in this region.

## 1 INTRODUCTION

All plant species have limits to the range of conditions in which they can live, and all but the narrowest endemics grow across environments that vary in biotic and abiotic conditions. This natural complexity has significant impacts on individual survival and reproduction, and thus plant evolution (Ackerly et al., 2000; Linhart & Grant, 1996; Loveless & Hamrick, 1984; Reich et al., 2003). As plants are subject to different conditions associated with their local environment, populations of the same species will experience differential selection pressures (Antonovics & Bradshaw, 1968; Clausen, Keck, & Hiesey, 1948; Langlet, 1971; Turesson, 1922), creating habitat‐correlated intraspecific variation. When this intraspecific variation results in populations that are more fit in their home environment than foreign populations, these populations are considered to be locally adapted (Blanquart, Kaltz, Nuismer, & Gandon, 2013; Kawecki & Ebert, 2004). The existence of local adaptation is well‐established across different organisms and ecosystems, although our synthetic knowledge of this important topic rests on surprisingly few reviews of the subject (Hereford, 2009; Leimu & Fischer, 2008; Oduor, Leimu, & van Kleunen, 2016). Here, we focus on a particular region and ask if plant species share patterns of intraspecific variation and local adaptation, and, across taxa, what functional traits and environmental variables are most important for such patterns in this region. The regional focus provides a strong test of expectations generated from more heterogeneous samples, facilitates comparison of the strength of selection among specific traits, and provides an opportunity to link basic evolutionary patterns with applied concerns.

The detection of local adaptation ideally involves reciprocal transplant experiments designed to test for a local advantage across environments (Blanquart et al., 2013; Bucharova, Durka, et al., 2017). However, patterns associated with local adaptation (hereafter, signatures) can be detected in nonreciprocal comparisons of different populations of the same species (Endler, 1986). When populations are locally adapted to environmental variables, we expect to see three basic signatures from common garden experiments: (a) differences among populations in fitness‐related traits, (b) correlations between these trait values and environmental or other habitat‐related variables, and, if reciprocal transplants have been conducted, (c) higher fitness of local over nonlocal populations in the local environment. Although population differences (signature 1) are necessary for local adaptation, they alone are not sufficient evidence due to factors such as genetic drift, high gene flow, and rapid environmental change, among other factors (Blows & Hoffmann, 2005; Kawecki & Ebert, 2004). While fitness differences in reciprocal transplant experiments (signature 3) are the “gold standard” for detecting local adaptation, there are experimental trade‐offs between the number of populations sampled and the ability to do fully reciprocal transplants (Blanquart et al., 2013). Thus, correlative approaches (signature 2) are popular alternatives that can sample many more populations to infer local adaptation (St Clair, Mandel, & Vance‐Borland, 2005), though spurious correlations, low sample sizes, or high variability in trait values could over‐ or underpredict the degree of local adaptation in wild populations using this approach. Given these considerations, separately reporting all three signatures can give an overall picture of the likelihood of within‐species variation and potential local adaptation in a region, and is the first step toward a better understanding of variation in the strength and consistency of natural selection (Siepielski, Dibattista, & Carlson, 2009).

The Great Basin Desert of North America is a ~540,000 km^2^ cold desert landscape characterized by hundreds of internally draining basin and range formations, which create high spatial and environmental heterogeneity and variability (Comstock & Ehleringer, 1992; Tisdale & Hironaka, 1981). While these are the kinds of conditions that would be expected to result in widespread local adaptation, the flora of the Great Basin is poorly represented in the relatively few reviews on the subject (Hereford, 2009; Leimu & Fischer, 2008; Oduor et al., 2016), and this has resulted in uncertainty as to the prevalence, magnitude, and importance that local adaptation plays in this large and increasingly imperiled region (Chivers, Jones, Broadhurst, Mott, & Larson, 2016; Jones, Monaco, & Rigby, 2015; United States House of Representatives (Committee on Appropriations), 2014). Gaining a better understanding of local adaptation in the Great Basin is important not only because it is a large, relatively intact floristic region in the Western United States, but also because this information has direct impacts on conservation and restoration efforts. Large‐scale, seed‐based restoration has been very common in the Great Basin for many decades (Pilliod, Welty, & Toevs, 2017), and trends in large destructive wildfires (Dennison, Brewer, Arnold, & Moritz, 2014) and other disturbances (Davies et al., 2011; Rowland, Suring, & Michael, 2010) ensure even higher demand for restoration efforts in the future. Guided by the various national policies and strategies dating from the 1960s (Richards, Chambers, & Ross, 1998) to the present National Seed Strategy (Plant Conservation Alliance, 2015) and Integrated Rangeland Fire Management Strategy (USDOI, 2015), a growing majority of these efforts are using native plants. However, few of the widely available sources of commercially produced seeds of native species originate from populations within the Great Basin (Jones & Larson, 2005) or have been selected based on their success in restoring Great Basin habitats (Leger & Baughman, 2015). Further, demand for native seed has always exceeded supply (Johnson et al., 2010; McArthur & Young, 1999), which has resulted in the prioritization of seed quantity and uniformity over population suitability and local adaptation (Leger & Baughman, 2015; Meyer, 1997; Richards et al., 1998). Therefore, it is still uncommon for restorationists in this region to prioritize or even have the option to prioritize the use of local populations, despite growing support of the importance of such practices (Basey, Fant, & Kramer, 2015; Espeland et al., 2017).

Though our understanding of the prevalence and scale of local adaptation in the Great Basin is far from complete, there is an abundant literature of peer‐reviewed studies on the plants native to this region spanning over 75 years that have directly measured trait variation between populations via laboratory, greenhouse, or field common gardens and reciprocal transplants. Many of these studies have also tested for correlations between intraspecific variation and environmental variables, and some were designed to detect local adaptation. This research includes studies of germination patterns (McArthur, Meyer, & Weber, 1987; Meyer, Beckstead, Allen, & Pullman, 1995), large genecology experiments (Erickson, Mandel, & Sorenson, 2004; Johnson, Leger, & Vance‐Borland, 2017), and reciprocal transplants (Barnes, 2009; Evans & Young, 1990), among other types of studies. This rich literature provides an opportunity to summarize local adaptation and its associated patterns, or signatures (defined above), in this region, as well as describe which phenotypic traits have the strongest signatures of local adaptation.

Here, we present results of a broad literature review and subsequent meta‐analysis using published studies that compared phenotypic traits of multiple populations of native Great Basin species in one or more common environments. Our first objective was to record published instances of the three expected signatures of local adaptation (population variation, trait‐by‐environment association, and greater local fitness) within grasses, forbs, shrubs, and deciduous trees native to the Great Basin, asking how common these signatures are, as well as which phenotypic traits and environmental variables were most commonly associated with these signatures. We also present results by taxonomic group, lifeform, lifespan, distribution, and mating system. This first objective encompassed all possible studies, including those that did not provide sufficient details for formal meta‐analysis, which allowed us to incorporate the broadest range of studies, including older studies that provided minimal quantitative detail. Our second objective was to examine links between the magnitude of trait and environmental divergence (mean annual precipitation and mean annual temperature) among populations across multiple taxa, for the subset of experiments amenable to this approach, asking whether populations from more similar environments were more similar in phenotypic traits. We also used meta‐analysis to ask which traits and environmental variables showed the strongest patterns of association.

We expected to find widespread evidence of local adaptation and its signatures in the plants of the Great Basin, and we hypothesize that phenological and size‐based traits, which show phenotypic variation in response to climate variation in both plants and animals (Anderson, Inouye, McKinney, Colautti, & Mitchell‐Olds, 2012; Sheridan & Bickford, 2011) and have been observed to be under selection in the Great Basin (Leger & Baughman, 2015), would be important indicators of adaptation in this region. We discuss our results both as a contribution to our general understanding of natural selection in plants, and as an example of evolutionary theory applied to the management and restoration of a large geographic region, where active and ongoing management can benefit from information on intraspecific variation and local adaptation.

## 2 METHODS

### 2.1 Literature search

We began by using the search engines Google Scholar and Web of Science to search for combinations of key terms (see additional methods in Appendix S1). In order to be included in our review, a study had to meet all these criteria:

1. Examined a species that is native within the floristic Great Basin
2. Examined and compared more than one population of that species
3. Measured at least one phenotypic, physiological, phenological, or other potentially fitness‐related trait (e.g., survival; hereafter, trait)
4. Measured the trait(s) of the populations in at least one common environment (including laboratories, growth chambers, greenhouses, or outside gardens; hereafter, garden).

A plant was determined to be native to the Great Basin if the taxa had at least one occurrence with native status within the floristic Great Basin according to occurrence information from the USDA Plants Database (USDA & NRCS, 2018) and/or the U.S Virtual Herbarium Online (Barkworth et al., 2018). A total of 170 studies published between 1941 and July 2017 were encountered that met these criteria.

### 2.2 Categorization and scoring of literature

All studies meeting our criteria were categorized and scored for each signature. The coordinates of all gardens and populations in each study were recorded or, if possible, generated from localities described in the studies (Appendix S1). For each study, we then noted these 15 characteristics: the year published, year(s) of plant material collection, year(s) of experimentation, number of years reported, taxa (genus, species, subspecies), life history traits (taxonomic status, lifeform, geographic range, life span, breeding system), experiment type (laboratory, greenhouse, common garden, reciprocal transplant), number of gardens, number of populations tested, which generation of material was used, and whether or not experimenters attempted to control for maternal effects prior to testing (Appendix S1). Life history traits were compiled for each taxon from the USDA Plants Database as well as from published literature (Appendix S1). Each taxon (subspecies level, if given) was entered separately for studies addressing multiple taxa. In studies where more than one experiment was performed, and the experiments differed in the experiment type (defined above), the identity of the populations being compared, and/or the generation of material used, they were entered as separate experiments. In cases where the list of tested populations was identical among multiple published studies, and these materials came from the same collections, these experiments were entered separately if the garden type or location(s) differed among the studies or if authors separately published different traits from the same gardens, ensuring that no trait was recorded twice for the same set of populations in the same garden. In cases where the list of tested populations did not completely overlap between studies, even if some from each study arose from the same collections, they were entered separately. These methods carefully emphasized the inclusion of the greatest number of relevant experiments and traits without duplication, but nonetheless resulted in some nonindependence between some experiments. A total 327 taxa‐specific entries (hereafter, experiments) were generated from the 170 published studies (Appendix S2).

The first two expected signatures of local adaptation were scored using a Yes/No designation for each experiment which considered all measured phenotypic traits. A score of “Yes,” or, in the absence of supporting statistical evidence, “Authors claim Yes,” was given when at least one measured trait significantly demonstrated the signature for at least two populations, and a score of “No” or “Authors claim No” was given when the signature was not detected between any pair of populations (Appendix S1). In addition, each of the measured and reported traits and environmental variables were scored (hereafter, trait scores) in the same way for each signature. Of the 327 experiments, 305 (93.3%) met the criteria to score for among‐population variation (signature 1) and 161 (49.5%) met the criteria to score for trait‐by‐environment association (signature 2). Pearson's chi‐squared tests were used to determine whether there were differences in signatures 1 and 2 among plants with different life history traits, using totals from both “Yes/No” and “Authors Claim Yes/No” results, excluding any life history groups represented by <10 experiments.

To score whether there was higher fitness of a local population in a common garden (hereafter, signature 3), only experiments in which outdoor reciprocal transplants or common gardens were performed using a local population in at least one garden were considered (Appendix S1). Additionally, the experiment had to measure a fitness‐relevant response: survival, reproductive output (number of seeds or flowers, or other reproductive output), a fitness index (a combination of several size and production traits), or total aboveground biomass. Each experiment was assigned a composite score to fully capture variation in the performance of each garden's local population, across multiple gardens as well as through multiple sampling dates (Appendix S1). The five possible composite scores were “Yes for all gardens at all times,” “Yes for all gardens at some times,” “Yes for some gardens at all times,” “Yes for some gardens at some times,” and “No for all gardens at all times.” These scores refer only to those gardens within each experiment that included their own local population. Of the 326 experiments, 27 (8.3%) were appropriate for this scoring. This scoring provides an estimate of the commonness of higher local fitness, but it is not a measure of the importance of the difference per se. For example, a fitness difference could occur uncommonly, but have a large impact on population trajectories (i.e., large differences in survival after a rare drought event).

Our dataset, which had uneven numbers of experiments representing each species, contained the possibility of bias associated with highly studied taxa influencing patterns more than less‐studied taxa. To ask how this affected overall results, we compared tallies of all scores without correcting for multiple experiments per species to tallies using an average score for each species for each signature. To generate these average scores for signature 1 and 2, we totaled all “Yes” and “Authors claim Yes” scores for each species and divided by the total number of scores (all Ys plus all Ns) for that species. For signature 3, all forms of “Yes” (all but “No for all gardens at all times”) were totaled into a Y and divided by the total number of scores. Then, we averaged these per species scores to re‐calculate overall effects in which each species was represented only once, and compared the results of the different averaging methods for each signature.

### 2.3 Quantitative comparison of trait‐by‐environment associations

As a complement to the survey of author‐reported results described above, we conducted a further, quantitative analysis of trait and climate values. Specifically, to examine associations between the differences in trait values and the differences in environmental and geographic distance among population origins, we utilized experiments from which population‐specific trait data and geographic coordinates could be extracted or obtained through author contact. Data from laboratory and greenhouse experiments were not considered for this extraction. First, we identified the most commonly measured traits across studies, which were then manually extracted from text, tables, or graphical data (Appendix S1). Next, we extracted trait data from the latest sampling date for which the most populations at the most gardens were represented, and if multiple treatments were used, we only extracted data for the author‐defined “control” treatment. However, if no control was defined, we used the treatment that was the most unaltered or representative of the garden environment (e.g., unweeded or unwatered). For each population/trait combination, we used either author‐provided mean values or calculated a mean trait value from available data. Rather than averaging values across gardens, data, data from each garden location within each experiment, were extracted separately and considered its own sample. We did this because it is not uncommon for traits to be expressed differently in different common garden locations (Johnson et al., 2017). Finally, we generated 30‐year annual precipitation and mean annual temperature values for each population's location of origin using the ClimateNA v5.10 software package based on methodology described by Wang, Hamann, Spittlehouse, and Carroll (2016). These 30‐year averages are calculated every 10 years (i.e., 1951–1980, 1961–1990). Because studies took place at many times over the last 75 years, we used the most proximate climate normal for each experiment that did not include or surpass the years during which the experiment's populations were collected (Appendix S2).

To reduce the likelihood of spurious correlations or false negative results, we limited this dataset to traits measured in at least 5 populations in at least 20 common garden locations (mean locations per trait: 34.4; range: 21–46), resulting in 81 locations (from 56 experiments) that measured at least one of eight frequently measured phenotypic traits (Table 1). Within each location, we calculated pairwise Euclidean distances for each trait value, climate factor, and geographic distance for every possible pair of populations. Geographic distances were generated using the earth.dist function in fossil package (Vavrek, 2011) in the statistical computing environment R (R Core Team, 2017). Then, partial Mantel tests were used to compare pairwise trait and climate distances for each experiment while controlling for geographic distances, using the vegan package (Oksanen et al., 2018) in R (R Core Team, 2017). We used the metacor.DSL function in the metacor package (Laliberté, 2011) to generate an overall effect size (partial correlation) and upper and lower confidence intervals for each combination of trait and environmental variable. Lastly, to better understand effect sizes for a subset of species, we ran simple linear regression analyses for each location, comparing average trait values and environmental values to generate a slope that estimated trait change per unit change in climate factors. Experiments with *R* ^2^ values of 0.2 or less were excluded from this particular analysis, and the median slope across experiments was retained as an estimate of the trait‐by‐environment relationship. The arbitrary cutoff (*R* ^2^ = 0.2) for this step was used simply as a way to focus on and report effect sizes from some of the stronger biological relationships that could be of particular interest to managers, restoration practitioners, and evolutionary ecologists. Due to limited sample sizes for factors such as lifeform, mating system, geographic distribution, we did not include these factors in any of the quantitative analyses, but present lifeform (shrub, grass, or forb) information for each trait response as additional results in the Appendix S3.

**Table 1:** Traits measured in outdoor common gardens or reciprocal transplants for at least 5 populations in at least 20 common garden locations, with data available from text, tables, author contact, or extraction from figures. Note that in some cases, multiple highly similar measures were grouped, as indicated in footnotes

Trait	Units	Locations
Date—flowering^a^	# days	34
Size—floral^b^	cm	22
Height—plant	cm	46
Size—leaf^c^	cm	30
Mass—shoots^d^	g	43
Number—inflorescence^e^	#	36
Number—seeds^f^	#	21
Survival	%	43

a Flowering date or any other floral phenology.

b Any size measurement of a floral structure.

c Most frequently, leaf length; occasionally leaf width.

d Any measure of aboveground biomass.

e Counts of flowers or flowering structures.

f Most frequently seed number, but also seed yield in mass and/or seed yield rating/rank.

## 3 RESULTS

### 3.1 Summary of reviewed literature

**Figure 1:**
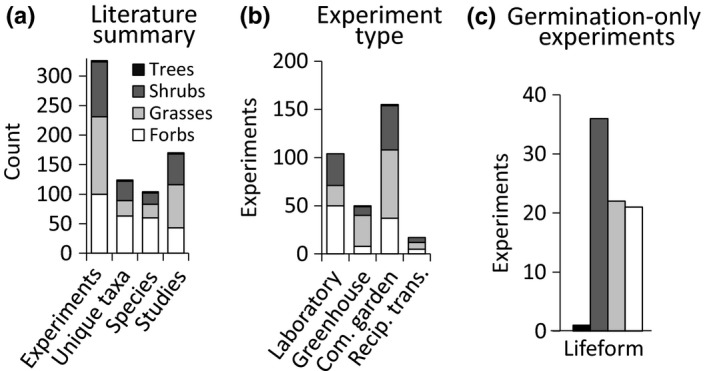
Summary of reviewed literature that compared traits among at least two populations in at least one common environment, by lifeform. Total counts of published studies, species, taxa, and taxa‐specific experiments (a); types of experiments (b); and total counts of experiments that measured only germination traits, (c)

**Figure 2:**
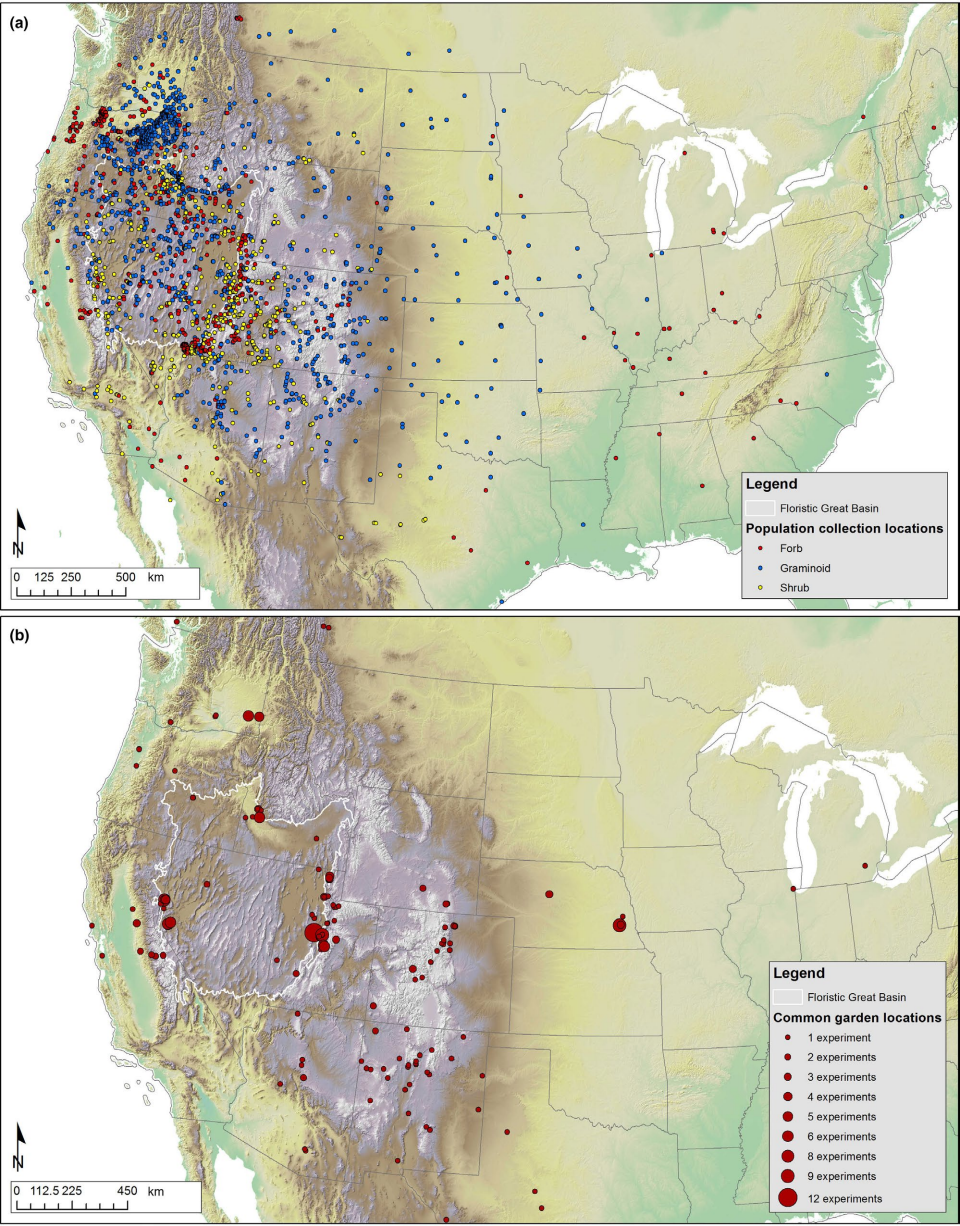
Map of 129 different outdoor common garden locations (a) and 2,953 unique population collection sites (b) for the 80% of outdoor gardens and 91% of experiments for which coordinates could be obtained or generated, from 170 studies reviewed. The size of the marker in panel (a) represents the number of experiments in which each specific garden location was used, with larger symbols indicating garden locations used in more experiments. Although all species represented are native to the floristic Great Basin (white outline), many populations were collected and tested outside this region

Our literature search revealed 170 published studies that measured trait responses from more than one population in at least one common environment, resulting in 327 separate experiments involving 121 taxa of 104 species of grasses, shrubs, forbs, and deciduous trees (Figure 1). These experiments represent approximately 3,234 unique populations tested in approximately 208 outdoor garden locations (Figure 2) and 154 indoor laboratory or greenhouse experiments. Grasses accounted for 21.0% of the taxa and 40.2% of the experiments, forbs composed 50.8% of the taxa and 30.7% of experiments, shrubs 26.6% of the taxa and 28.5% of experiments, and deciduous trees accounted for only 1.6% of taxa and 0.6% of experiments (Figure 1a). Experiments were most commonly conducted in nonreciprocal outdoor common gardens (47.5%) or in the laboratory (31.9%), with fewer conducted in greenhouses (15.3%) or in reciprocal outdoor gardens (5.2%, Figure 1b). For experiments in outdoor gardens, the median number of gardens per experiment across lifeform ranged from 1 (grasses, shrubs, and trees) to 2 (forbs) for nonreciprocal gardens, and from 2 (grasses and forbs) to 4 (shrubs) for reciprocal gardens. Overall, the median number of populations tested in each experiment was 5 (range = 2–193, IQR = 3–11.5, Figure 1c) and was slightly lower for shrubs (median = 4, range = 2–111, IQR = 2–8) than grasses (median = 6, range = 2–193, IQR = 3–12.25), forbs (median = 6, range = 2–67, IQR = 3–10.25), and trees (median = 7, range = 5–9, IQR = 6–8).

**Figure 3:**
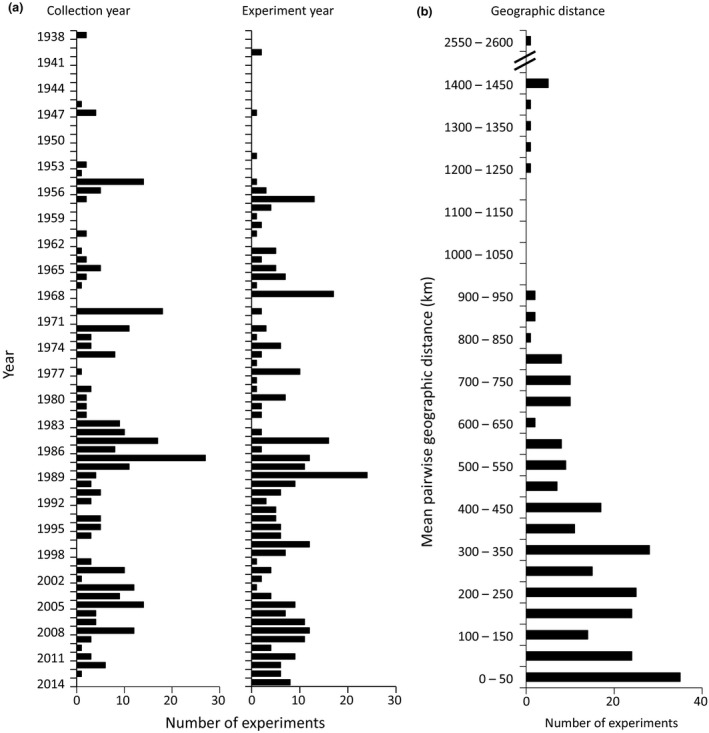
Summary of the years in which the collections of each experiment were made (a, left), the year each experiment was performed (a, right), and the average geographic distance among population collections sites in each experiment. The percent of 327 experiments that reported this information were 99% and 88% (respectively) for panel a, and 80% for panel b. Collection year and experiment year represent the average for each experiment, as it was common for materials to be collected and tested over multiple years for each experiment. Geographic distance is the mean pairwise distance among populations in each experiment; note the noncontinuous vertical axis

**Figure 4:**
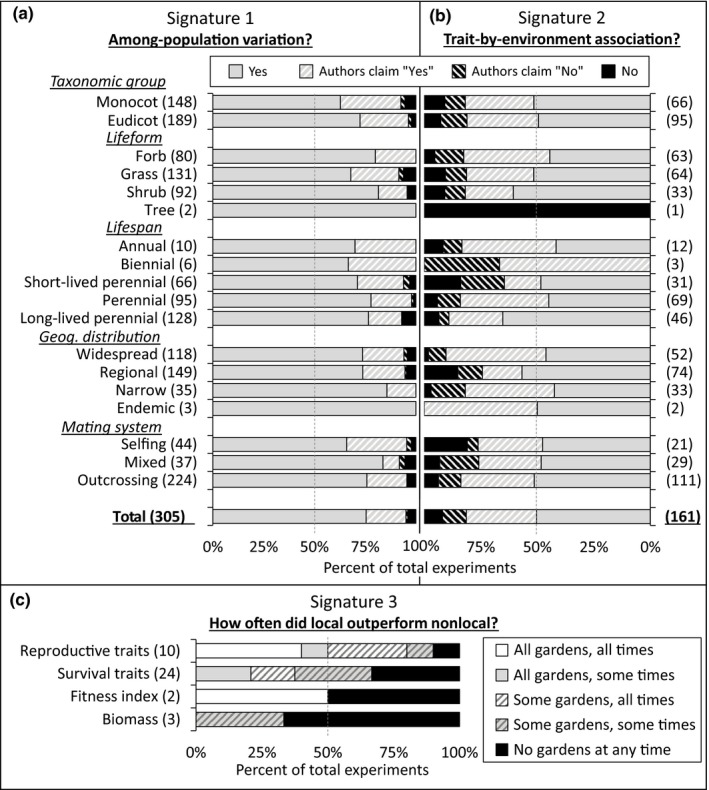
Summary of among‐population variation (a, signature 1) and trait‐by‐environment associations (b, signature 2) for any measured trait, grouped by five life history traits. Summary of local advantage (c, signature 3) for reproductive traits, survival traits, fitness indices, or biomass. Data compiled from 327 experiments from 170 published studies on Great Basin plants (see Appendix S2 and available datasets in electronic supplementary material). For signatures 1 and 2, “Yes” and “No” represent statistical comparisons, while “Authors claim ‘Yes’” and “Authors claim ‘No’” represent textual, claim‐based results where supporting statistics were not reported (common in older studies). For signature 3, most experiments had multiple gardens, and many evaluated performance at multiple sampling dates, leading to 5 different scores. These scores, from “All gardens, all times” to “No gardens at any time,” represent a gradient of incidence and frequency of this signature (see Methods). For all panels, numbers in parentheses, (*x*), indicate the number of experiments scored in a given category, and the dashed gray lines indicate 50%

Experiments took place between 1940 and 2015, with collections from native stands occurring between 1938 and 2013 (Figure 3a). One quarter of the experiments (24.5%) reported only early germination and seedling stages of plants (generally <0.5 years), while the remaining experiments (75.5%) reported study periods ranging from 0.5 to 17 years, with an average of 2.1 years (Figure 3b,c). Average pairwise geographic distance among populations per experiment for the 91% of experiments for which coordinates were available was 351 km ± 20 *SE*, with a range from 610 m to 2,551 km. Most experiments were conducted on taxa with regional distributions, perennial species, grasses, and outcrossing species; very few annuals, endemic species, or selfing species were represented (Figure 4). Over half of experiments (58.6%) tested plants grown directly from wild‐collected seeds (or the seed of wild‐collected adults), 16.9% tested wild‐collected adults, 13% tested materials with mixed generations since collection, 6.7% tested 1st or 2nd generation descendants of wild‐collected seeds, 0.3% tested only cultivars, and 4.3% did not provide enough information to determine.

### 3.2 Among‐population variation

Of the 305 experiments appropriate for addressing among‐population trait variation (signature 1), 290 (95.1%) experiments reported finding variation among populations in at least one phenotypic trait, with 230 (75.4%) of these 290 reporting significant variation, and 60 (19.6%) claiming such variation in the absence of any supporting statistics (Figure 4a). Only 12 (3.9%) experiments reported no such differentiation in any trait after statistically testing for it, and 3 (1%) claimed no such variation without presenting statistical evidence. When categorized by basic life history traits, several differences appeared among groups. Eudicots exceeded monocots (the majority of which were grasses) in the degree of population differentiation($X_{1}^{2}$ = 7, *p* = 0.0081), and, similarly, forbs and shrubs had more population differentiation than grasses ($X_{2}^{2}$ = 8.05, *p* = 0.0143). There were no significant differences in signature 1 among plants with different geographic distributions, life span, or breeding systems.

**Figure 5:**
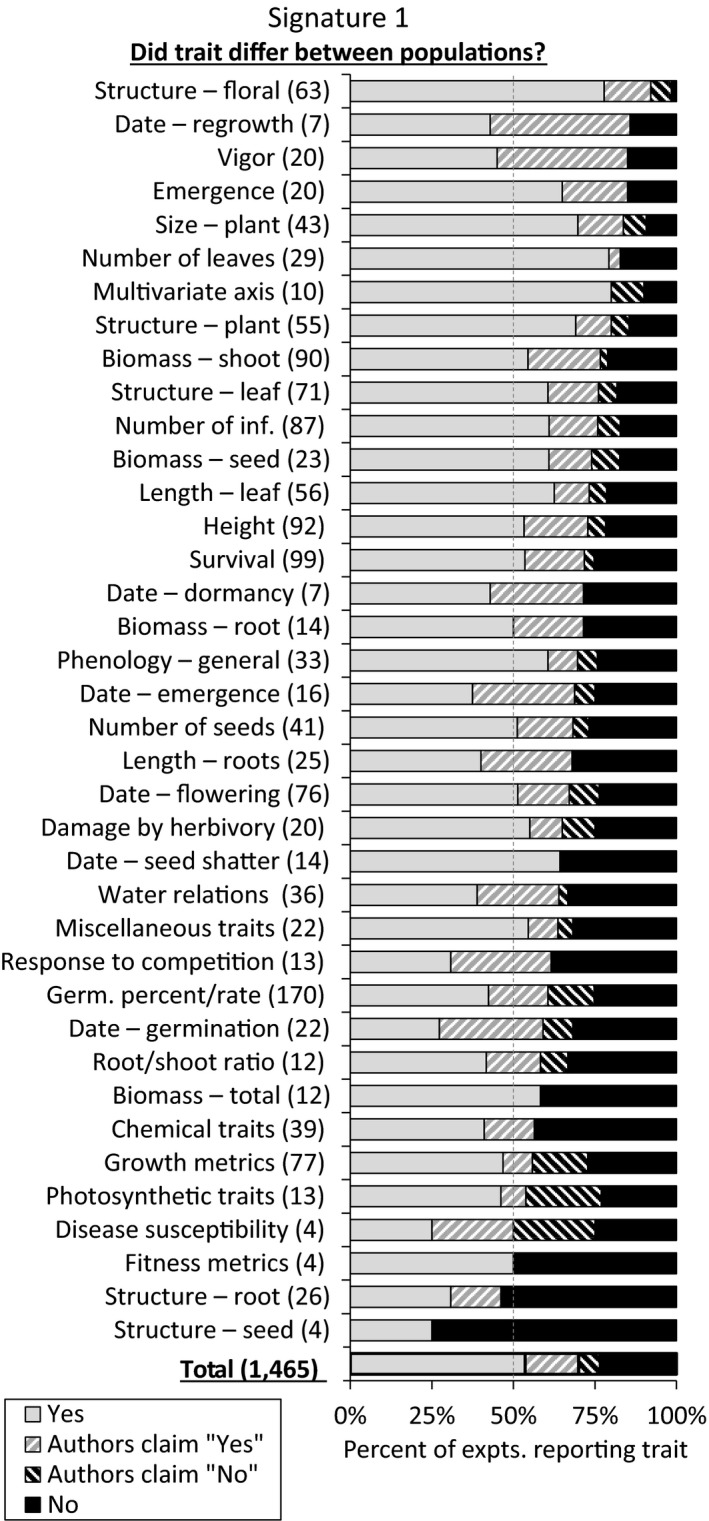
Summary of 1,465 trait scores from the 305 experiments appropriate for detecting signature 1 (differences between populations). Scores of “Yes” and “No” were supported by statistical comparisons, while the “Authors claim…” scores represent textual, claim‐based results where supporting statistics were not reported (common in older studies). Numbers in parentheses, (*x*), indicate the total experiments that measured each trait or reported each factor, and dashed gray line indicates 50%

A total of 1,465 trait scores were recorded from the 305 experiments appropriate for addressing signature 1. Frequently measured traits (20 or more experiments) that had differences between populations in over 75% of experiments (with or without supporting statistics) were floral structure, vigor, emergence, plant size, number of leaves, plant structure, shoot biomass, leaf structure, and number of inflorescences (Figure 5).

### 3.3 Trait‐by‐environment associations

Of the 161 experiments appropriate for testing trait‐by‐environment associations (signature 2), 131 (81.4%) reported associations for at least one comparison, with 81 (50.3%) supported by statistical tests and 50 (31.1%) supported by claims in the absence of statistics (Figure 4b). Conversely, 13 (8.1%) of experiments reported no such correlations after having statistically tested for it, and 17 (10.6%) reported no such correlations but lacked any supporting statistics. There were no significant differences in the commonness of trait‐by‐environment associations for taxonomic status, lifeform, geographic distribution, or breeding system, but perennials (both long‐lived and short‐lived) had more frequent correlations between traits and environment than did annuals or short‐lived perennials ($X_{3}^{2}$ = 8.08, *p* = 0.0444).

**Figure 6:**
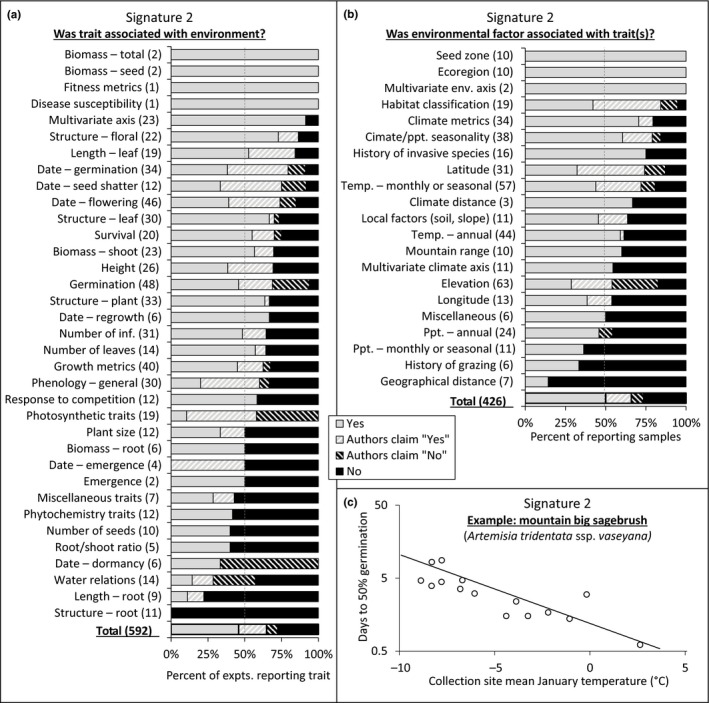
Summary of scores for associations between 592 traits (a) and 426 environmental factors (b) from the 161 experiments appropriate for detecting signature 2 (trait‐by‐environment association), expressed by trait/factors, and an example from the literature (c, redrawn with permission from (Meyer & Monsen, 1991)) in which date of germination for mountain big sagebrush is correlated with a measure of monthly temperature (treatment: 2‐week chill). Scores of “Yes” and “No” were supported by statistical comparisons, while the “Authors claim…” scores represent textual, claim‐based results where supporting statistics were not reported (common in older studies). For panels (a) and (b), numbers in parentheses, (*x*), indicate the total experiments that measured each trait or reported each factor, and the dashed gray lines indicate 50%

A total of 592 trait scores were recorded from the 161 experiments appropriate for addressing signature 2 (Figure 6a). Frequently measured traits (20 or more experiments) that were correlated with environmental variables in over 75% of experiments (with or without supporting statistics) were multivariate trait axes, floral structure, and germination date. Every remaining trait that was measured in >15 experiments was correlated with environmental characteristics in over 50% of experiments, and many, including leaf length, survival, flowering date, and leaf structure, were correlated with environmental variables in ≥70% of experiments.

A total of 426 environmental variable scores were recorded from the 161 experiments appropriate for addressing signature 2 (Figure 6b). Of the variables most frequently reported as correlated with plant traits, many categorical variables or composite metrics made this list, with seed zones, ecoregions, multivariate environmental axes, and habitat classifications topping the list of important environmental variables (important in >84% of experiments that reported them). Additionally, derived climate metrics (such as climate continentality, heat/moisture index, and potential evapotranspiration), climate seasonality, and history of invasive species presence were correlated with plant traits in over 75% of studies that reported them.

### 3.4 Higher local performance in a local common garden

The 27 experiments that were suitable for detecting higher fitness of a local population in a local garden (signature 3) generated 39 scores (some experiments measured multiple fitness traits), with 27 scores (69.2%) reporting signature 3 for at least one fitness trait in at least one of the tested gardens during at least one sampling date, and the remaining 12 scores (30.8%) not reporting signature 3 at any point (Figure 4c). Thirty‐two of the 39 scores (82%) were generated from experiments with more than one garden. Survival was the most frequently measured fitness trait in these experiments, reported in 24 of the 27 experiments, followed by reproduction (10), biomass (3), and fitness indices (2). Incidence of the local‐does‐best pattern was highest in experiments that directly measured reproductive output, with 90% reporting higher values for locals at some point in an experiment, followed by survival (67%), fitness indices that incorporated biomass (50%), and biomass measures (33%). For experiments in which only “some” gardens showed local‐does‐best patterns (Figure 4c, hashed bars), the percentage of gardens showing this trend was 40%, 50%, and 40% for reproduction, survival, and biomass traits, respectively (not shown). For experiments in which only “some” sampling dates showed local‐does‐best patterns (gray bars), the percentage of sampling dates showing this trend was 56%, 47%, and 25% for reproduction, survival, and biomass traits, respectively (not shown).

### 3.5 Considering possible biases: highly studied species and maternal effects

The number of experiments per species in our dataset ranged from 1 (52 species) to 25 (*Artemisia tridentata*), with a median of 1 (IQR = 1–4). The most highly represented species were *Artemisia tridentata* (25 experiments), *Elymus elymoides* (24), *Ericameria nauseosa* (17), *Achnatherum hymenoides* (17), *Krascheninnikovia lanata* (13), *Pascopyrum smithii* (11), *Atriplex canescens* (9), *Leymus cinereus* (9), and *Poa secunda,* (8). Results in which scores were averaged for each species (see Methods) were similar to uncorrected results: Signature 1 was 4% higher when corrected (98% vs. 94%), signature 2 was 1% lower when corrected (79% vs. 80%), and signature 3 was 8% higher when corrected (78% vs. 70%). Thus, uncorrected calculations were used throughout our study.

Only 19 experiments (5.8%) used an experimental design that could control for maternal effects (e.g., growing all populations for a generation in a common environment before initiating an experiment). An additional 30 experiments (9.2%) were unclear on this point, and the remaining 278 (85%) experimented directly on populations differing in maternal environment. The incidence of population differences (signature 1) was 100% in the 16 experiments that moderated maternal effects, 95% for the 259 that did not make an attempt, and 97% for the 30 which were unclear. Too few of the experiments that attempted to control for maternal effects were appropriate for measuring signature 2 (4 experiments) and signature 3 (1 experiment) to compare incidences of these signatures.

### 3.6 Quantitative comparison of trait‐by‐environment associations

**Figure 7:**
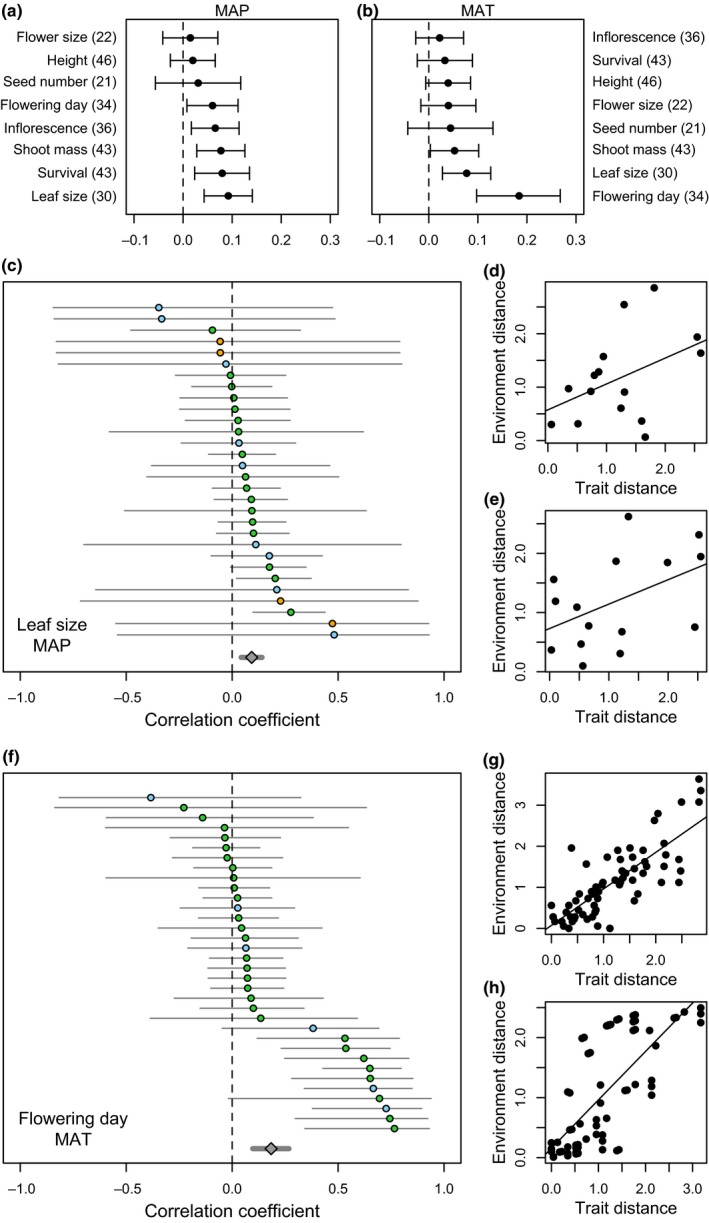
Results of comparisons of pairwise trait and environmental distances for eight frequently measured phenotypic traits and (a) the mean annual precipitation (MAP) or (b) mean annual temperature (MAT) at the original collection location. Values are effect sizes and 95% confidence intervals for each trait, averaged across all experiments for which data were available (number of experiments in parentheses). Examples of the two strongest relationships are shown for leaf size and MAP (c), where each line shows the correlation coefficient and confidence intervals for an individual experiment, for which we calculated the relationship between differences in percent survival and difference MAP at location of origin. Color indicates functional groups: Green = grasses, blue = shrubs, orange = forbs. Examples are shown for the two highest effect sizes: d), experiment 297A, (Kramer, Larkin, & Fant, 2015), *Penstemon deustus* and e), experiment 297A, (Kramer et al., 2015), *Eriogonum microthecum*. Similarly, flowering time and MAT (f) is shown, with examples of g) experiment 271A, (Larsen, 1947), *Schizachyrium scoparium*, and h) experiment 245A, (Ward, 1969), *Deschampsia caespitosa*. Full results for each trait/environment relationship are shown as additional results in Appendix S3

Overall, we found positive relationships between the magnitude of differences among populations in all eight phenotypic traits and the magnitude of differences between MAT and MAP at the collection locations (Figure 7). The strongest relationship was observed between differences in flowering time and differences in MAT, and leaf size also showed a strong relationship with MAT. Multiple strong relationships were observed between trait/environment divergence for MAP, with leaf size, survival, shoot mass, inflorescence number, and flowering time all showing strongly positive relationships for grasses, forbs, and shrubs. (Figure 7 and Appendix S3). Regression analyses demonstrated that, for the 15 common garden locations in which strong flowering time and MAT relationships were observed, each degree change in MAT was associated with a median change of 3.5 days (IQR = 1.2–5.3) in flowering time. Small sample sizes (few experiments that could be included in the analyses) and challenges with interpreting changes in physical traits across species of various shapes and sizes precluded the presentation of estimates of this nature for the other trait‐by‐environment relationships.

## 4 DISCUSSION

Our results represent the most extensive review of intraspecific variation and local adaptation for plants native to the floristic Great Basin, a region comprised of largely continuous but increasingly imperiled arid and semi‐arid plant communities (Davies et al., 2011; Finch et al., 2016). Additionally, they represent a significant addition to the noteworthy though relatively small number of reviews investigating this topic in a manner that identifies individual traits and environmental factors involved. We found that Great Basin plant species contain large amounts of intraspecific diversity in a wide range of phenotypic traits, that differences in these phenotypic traits are often associated with the heterogeneous environments of origin, and that differences among populations are commonly relevant to outplanting fitness. The cascading importance of intraspecific variation for the structure, functioning, and biodiversity of communities and ecosystems can be considerable (Bolnick et al., 2011; Bucharova et al., 2016) and may equal or exceed the importance of species diversity (Des Roches et al., 2018). Our quantification of local adaptation and trait–environment associations should serve as encouragement to seriously consider intraspecific diversity in native plant materials used in restoration and conservation in this region throughout the selection, evaluation, and development process (Basey et al., 2015). The results reported here should also serve as a cautionary note to restoration approaches that focus on only a few specific traits or search for general‐purpose genotypes. Our results suggest that, in the absence of species‐specific information to the contrary, it is reasonable to assume that local adaptation is present in this region, and that locally sourced populations would outperform nonlocal populations a majority of the time.

Our investigation encompassed 170 studies published between 1941 and 2017 in which over 3,230 unique populations of 104 native Great Basin plant species were compared in 327 experiments, ranging from laboratory germination trials to multiple‐year common gardens and reciprocal transplants. The great majority (95%) found differences between populations (signature 1) in the majority of traits measured in a common environment, which indicates that different traits are variable among populations, at both small and large geographic scales. Additionally, a clear majority (81.4%) of experiments found trait‐by‐collection environment associations (signature 2), suggesting that intraspecific variation is frequently an adaptive outcome of natural selection in heterogeneous environments (Linhart & Grant, 1996; Reich et al., 2003). In experiments suitable for detecting local performance advantages (signature 3), local populations had higher performance (measured by differences in reproductive output, survival, and biomass) than nonlocal populations more often than not (69.2%), and this was particularly true when researchers reported traits related to reproductive output (90%). We used a vote‐counting method to summarize results for our broadest pool of studies, allowing us to incorporate a wealth of older studies for which quantitative details were not available. Results from a vote‐counting approach can sometimes differ from results of meta‐analysis, as vote‐counting does not incorporate the same level of detail about factors such as study size or effect size (Combs, Ketchen, Russell Crook, & Roth, 2011). However, in our study, the overall incidence of “local does best” in the Great Basin is similar to other reviews that have found local adaptation to be commonplace, but not ubiquitous. In a review of local adaptation in plants that compared survival, reproduction, biomass, and germination traits in reciprocal transplants, Leimu and Fischer (2008) found that local plants outperformed nonlocal ones in 71% of 35 published experiments. Similarly, Hereford (2009) quantified local adaptation in 70 published studies (50 of them plants), reporting only survival or reproductive traits, and found evidence of local adaptation in 65%–71% of experiments. Our results indicated that the strongest indication of local adaptation came from experiments that directly measured reproductive output, and that using biomass as a fitness proxy may not be an effective way to compare relative performance in the Great Basin. This is consistent with a previous study that demonstrated selection for smaller, rather than larger, individuals in disturbed arid systems (Kulpa & Leger, 2013). Literature reviews conducted across biomes may occlude regionally important trait differentiation and mask patterns of local adaptation, as we might expect, for example, biomass to be more strongly linked to fitness in regions where light is a contested resource (Espeland, Johnson, & Horning, 2017).

There are many processes that can reduce or prevent the development of local adaptation, such as the lack of divergent selection between sites, high gene flow, rapid or extreme environmental change, high phenotypic plasticity, and/or low genetic diversity (Blows & Hoffmann, 2005; Kawecki & Ebert, 2004; Sultan & Spencer, 2002). The high incidence of intraspecific variation, much of it habitat‐correlated, that we found in the literature confirms that divergent selection by heterogeneous environments is the norm for species native to the Great Basin, presumably outweighing the balancing effects of gene flow and genetic drift. Key environmental factors in the Great Basin such as fire frequency, grazing regimes, resource availability, and climate are certainly being altered to varying degrees by invasive species introductions, changing land uses, and climate change, and it can be argued that such changes could outpace the ability of local populations to remain adapted to their surroundings (Breed, Stead, Ottewell, Gardner, & Lowe, 2013; Havens et al., 2015; Jones & Monaco, 2009; Kilkenny, 2015). However, our analysis also demonstrated relatively high instances of trait correlations with relatively recent disturbances such as invasive species introductions. Rapid evolution in response to invasive species (Oduor, 2013) and other anthropogenic changes (Franks, Weber, & Aitken, 2014; Hoffmann & Sgrò, 2011) has been documented for many species, indicating that local adaptation can evolve rapidly in some circumstances.

Some traits and environmental characteristics stood out as particularly important indicators of local adaptation and its signatures across the studied taxa. For example, in our quantitative comparison of divergence in traits and environments, flowering phenology was strongly affected by MAT, with a median change of 3.5 days in flowering time per degree change in MAT of collection origin. Flowering phenology, along with germination phenology, were also in the top tier of frequently measured traits that showed significant correlations with environmental variables, consistent with other studies that have shown reproductive (Bucharova, Michalski, et al., 2017) and germination (Donohue, Brewer, Arnold, & Moritz, 2010) phenology to be an important response to environmental variation. Leaf size is also an important adaptive response to differences in temperature globally (Wright et al., 2017), and in concert with this, we saw overall positive responses to MAP and MAT for leaf size in our analyses as well as frequent trait‐by‐environment associations in the literature. Floral structure, which has important adaptive significance for angiosperms (Armbruster, 2014; Harder & Barrett, 2007), was among the most frequent traits scored for among‐population variation and trait‐by‐environment interactions. Seasonality of precipitation, which varies in this region depending on summer rainfall (Comstock & Ehleringer, 1992), was more predictive of trait variation overall than was mean annual precipitation (signature 2). In our quantitative comparisons, differences in MAP values were important for multiple phenotypic traits, including leaf size, shoot mass, reproductive output, and flowering phenology, in addition to being important for overall plant survival. Larger scale environmental descriptors, such as ecoregions and seed transfer zones, universally demonstrated signature 2, likely because they were developed based on climate/soil/vegetation associations or, in the case of seed transfer zones, developed based on trait‐by‐environment correlations. As found in other reviews (Geber & Griffen, 2003), physiological traits, phytochemical traits, and root traits were not measured as frequently as other traits, and though these did not show as frequent associations with environmental characteristics as other traits, they are known to vary across environments in some systems (Reich et al., 2003). Additional studies of these traits in the Great Basin would be informative and could reveal different patterns than those observed here.

As in any review and analysis of published papers, there are elements of our design that were difficult to control. For example, consistent with other reviews (Gibson, Espeland, Wagner, & Nelson, 2016), the vast majority of studies involved wild‐collected plants or seeds, and thus maternal environment effects almost certainly affected some results (Bischoff & Müller‐Schärer, 2010; Espeland, Perkins, Johnson, & Horning, 2016). Additionally, though the majority of populations tested in the literature were from western states, some of the populations compared in the literature were collected from well outside of the Great Basin, which increased the likelihood of observing local adaptation in these species. However, understanding patterns of intraspecific variation across the full range of the species native to the Great Basin is pertinent because it has been common (and for some species, ubiquitous) to utilize sources of native species originating from outside the Great Basin to use for restoration within the Great Basin (Jones & Larson, 2005). Finally, the scores and percentages for each of the signatures used throughout this study are uncorrected for phylogeny, as is our pairwise trait/environment analysis, and calculated such that each experiment is weighed equally. This introduces the possibility for phylogenetic biases, in which closely related taxa represented by many experiments affect the results more than less frequently studied taxa or groups of taxa. Though we did not conduct phylogenetic corrections for relatedness among taxa (Harvey & Pagel, 1991; de Bello et al., 2015), our results were essentially identical for signatures 1–3 when we averaged results across species (scores differed by +3%, −1%, and +8%, respectively), suggesting that our lack of phylogenetic corrections are not unduly affecting our results. We present all species‐specific information in Appendix S2 and available datasets section of the electronic supplementary material for further review.

Current approaches to seed sourcing in restoration and conservation include genetic (Williams, Nevill, & Krauss, 2014), genecological (Johnson et al., 2017), local‐only (Erickson et al., 2017), predictive (Prober et al., 2015), and agronomic (United States. House of Representatives. Committee on Appropriations., 2014)) strategies, as well as strategies mixing several of these viewpoints (Breed et al., 2013; Bucharova et al., 2018; Havens et al., 2015; Rice & Emery, 2003; Rogers & Montalvo, 2004). These approaches vary in the degree to which they meet the needs of seed producers and land managers while balancing population differences that stem from adaptive evolution in different environments. The prevalence of local adaptation and its signatures found in our study justify and support incorporating existing best practices (Basey et al., 2015; Espeland et al., 2017) for capturing and preserving important intraspecific variation into seed sourcing and plant production systems. For example, our results demonstrated a strong relationship between flowering time and MAT, so it would be wise to collect materials for research, evaluation, and testing from populations that vary in MAT, to collect seeds at multiple times to fully capture population variation in flowering time, and ensure that seeds are not transferred during restoration among sites that differ strongly in these characteristics. On the production side, best practices for seed harvesting should include methods that avoid inadvertent selection on flowering time, either for reduced variation or for a directional shift away from the wild condition. Similarly, emergence date was correlated with environmental variation in many plants, so testing in common gardens should involve seeding trials in place of or in addition to using transplants, and evaluation trials should guard against inadvertent selection on emergence timing by randomly, rather than systematically, selecting individuals to use in transplant experiments. These examples are not exhaustive, but demonstrate how evidence revealed by this study regarding which traits and environmental factors are generally involved in adaptation in this region can be used to improve approaches to seed sourcing and restoration. Finally, we acknowledge that ours is not the first review and meta‐analysis to affirm an abundance of intraspecific variation and local adaptation in plants. However, our focus on the Great Basin is important, because the large and frequent yet commonly unsuccessful restoration efforts occurring in this region have lagged behind those of other regions with respect to recognizing the importance of intraspecific variation and local adaptation on outplanting success.

## 5 CONCLUSIONS

Reestablishing and maintaining native plant communities in arid regions has proven challenging (Svejcar, Boyd, Davies, Hamerlynck, & Svejcar, 2017), and the lack of practical knowledge guiding more appropriate selection of seed sources is a major barrier (Friggens, Pinto, Dumroese, & Shaw, 2012; Gibson et al., 2016). The forestry industry has long adopted the principles of local adaptation in their reforesting guidelines with great success (Aitken & Bemmels, 2016; Johnson, Sorensen, St Clair, & Cronn, 2004; Matyas, 1996), and similar approaches to restoration in the rangelands of the Great Basin may also increase success as our data support similarly high levels of population differentiation within grass, forb, and shrub life history groups. Our results, including both a qualitative literature survey and a quantitative meta‐analysis, could benefit from future work using additional techniques to explore spatial structure (Griffith & Peres‐Neto, 2006) and the relative importance of geographic distance and environmental variation, especially as additional studies become available in the literature. Nevertheless, our results as they currently stand are in agreement with observations of abundant local adaptation in plant populations world‐wide, and further, we identified particular phenotypic traits (flowering and germination phenology, floral structures, leaf size, biomass, survival, and reproductive output), environmental characteristics (MAT, MAP, climate metrics, seasonality), and habitat classifications and site history (seed zones, ecoregions, history of invasive species) that were important predictors of local adaptation in plants native to the Great Basin floristic region. Given the speed and severity with which natural communities are being altered by anthropogenic factors, the application of an evolutionary perspective to restoration ecology is more important than ever. Adjusting seed‐selection priorities to account for the existence of locally adapted, intraspecific variation in the Great Basin will promote the maintenance and recovery of resilient, self‐sustaining vegetation communities in this region (Broadhurst et al., 2008; Lesica & Allendorf, 1999; Meyer, 1997; Rogers & Montalvo, 2004; Vander Mijnsbrugge, Bischoff, & Smith, 2010).

## CONFLICT OF INTEREST

None declared.

## AUTHOR CONTRIBUTIONS

EAL, OWB, FFK, EKE, RF, TNK, and JBSC conceived and designed the study; OWB conducted the literature search; OWB, ACA, FFK, JO, RCJ, and JBSC categorized, compiled, and extracted data; OWB, EAL, FFK, ACA, and MLF analyzed data; OWB, EAL, and ACA drafted the manuscript; all authors critically revised the manuscript for important intellectual content and approved of the version to be published.

## DEDICATION

We would like to dedicate this paper to the memory of our co‐author Dr. Erin K. Espeland, friend and collaborator to all of us, who worked on this manuscript. Erin's light and life will never be forgotten by those who knew her, and we want to recognize her creative contributions to the field of plant ecology, including this effort. Erin is dearly missed.

## Data Availability

Raw datasets and statistical code supporting this study (Baughman et al., 2019) have been deposited at Dryad, (https://doi.org/10.5061/dryad.3pf2cb4).
